# Pattern of initiation of monomorphic ventricular tachycardia in recorded intracardiac electrograms

**Published:** 2005-10-01

**Authors:** Majid Haghjoo, Arash Arya, Mohammad Ali Sadr-Ameli

**Affiliations:** Department of Pacemaker and Electrophysiology, Rajaie Cardiovascular Medical and Research Center, School of Medicine, Iran University of Medical Sciences, Tehran, Iran

**Keywords:** ventricular tachycardia, implantable defibrillator, intracardiac electrogram, initiation pattern

## Abstract

**Background:**

By analyzing stored intracardiac electrograms during spontaneous monomorphic ventricular tachycardia (VT), we examined the patterns of the VT initiation in a group of patients with implantable cardioverter defibrillators (ICDs).

**Methods:**

Stored electrograms (EGMs) were monomorphic VTs and at least 5 beats before the initiation and after the termination of VT were analyzed. Cycle length, sinus rate, and the prematurity index for each episode were noted.

**Results:**

We studied 182 episodes of VT among 50 patients with ICDs. VPC-induced (extrasystolic initiation) episode was the most frequent pattern (106; 58%) followed by 76 episodes (42%) in sudden-onset group. Among the VPC-induced group, VPCs in 85 episodes (80%) were different in morphology from subsequent VT. Sudden-onset episodes had longer cycle lengths (377±30ms) in comparison with the VPC-induced ones (349±29ms; P= 0.001). Sinus rate before VT was faster in the sudden-onset compared to that in VPC-induced one (599±227ms versus 664±213ms; P=0.005). Both of these episodes responded similarly to ICD tiered therapy.     There was no statistically significant difference in coupling interval, prematurity index, underlying heart disease, ejection fraction, and antiarrhythmic drug usage between two groups (P=NS).

**Conclusion:**

Dissimilarities between VT initiation patterns could not be explained by differences in electrical (coupling interval, and prematurity index) or clinical (heart disease, ejection fraction, and antiarrhythmic drug) variables among the patients. There is no association between pattern of VT initiation and the success rate of electrical therapy.

## Introduction

Sustained monomorphic ventricular tachycardia (VT) occurs as consequence of many heart diseases affecting ventricles such as coronary artery disease (CAD), congestive heart failure (CHF), myocardial infarction (MI), and long QT syndrome and accounts for most cases of sudden cardiac death [1]. So emergent treatment of it is of paramount importance in cardiology practice. Reentry, triggered activity, or abnormal automaticity are mechanisms thought to be responsible for the genesis of VT [2,3]. The contribution of each mechanism depends on the presence of underlying arrhythmogenic substrate and dynamic factors such as myocardial ischemia and autonomic influences [4-6]. In the setting of CAD or previous MI, reentry is more common [2,7-9]. Recognition of specific electrogram patterns occurring at time of VT initiation can help to understand the electrophysiologic mechanism responsible for arrhythmia initiation and may lead to better diagnostic and therapeutic intervention.

Previous studies analyzed the mechanism of VT based on retrospective evaluation of 24-hour holter monitoring [10-14]. They are limited by the accidental registration of arrhythmia episode, the small number of patients included and the fact that each patient is only registered once. An extended recording of electrical events surrounding delivered and aborted ICD therapy not only permits more accurate characterization of the rhythm leading to device intervention, but also provides documentation of electrical events immediately before index arrhythmia [15-18].

This study was undertaken to evaluate the pattern of initiation of monomorphic VT by analyzing stored intracardiac EGMs from patients with ICDs. We, specially, tried to relate the initiation pattern of first episode with subsequent VT. We also sought to correlate the presence of various clinical factors and arrhythmia characteristics such as cycle length with different patterns of VT initiation. Finally, we determined the success rate of ICD therapy in two VT initiation groups.

## Materials and Methods

### Study population

All episodes of VT in patients with ICD implanted at our center between January 1999 and June 2001 were reviewed. Intracardiac stored EGMs with spontaneous sustained VT requiring therapy with antitachycardia pacing or direct-current cardioversion were identified. Only events that were monomorphic and had a minimum of 5 beats before the onset and after the termination of VT were selected for further analyses.

### Study protocol

Monomorphic VT was identified by a sudden increase in rate along with a change in electrogram morphology from the baseline rhythm, a constant cycle length that did not vary > 10% and uniform electrogram morphology during the tachycardia. A ventricular premature complex (VPC) was identified as any electrogram preceding the VT with morphology different from that of the baseline rhythm and coupling interval < 90% of the sinus cycle length. The first beat of VT, when morphologically different from subsequent tachycardia, was considered a VPC. In cases in which the first beat of VT was morphologically similar to the subsequent tachycardia, the coupling interval between the first beat and the VT was evaluated. That beat was considered either (1) the first beat of VT when the coupling interval was <110% of VT cycle length, or (2) a VPC if the coupling interval was >110% of the VT cycle length. VT episodes were categorized into 2 groups depending on the type and morphology of the last 5 beats before the initiation of VT: (1) sudden onset without preceding VPC, (2) Extrasystolic onset (VPC-induced) with preceding VPCs.

Premature depolarizations were recorded as similar or dissimilar to the subsequent VT based qualitatively on electrogram morphology. The cycle length of all beats during baseline rhythm and VT was measured. Mean sinus rate was taken as the average of all sinus beats before VT.

Prematurity index was calculated by normalizing the coupling interval to the preceding RR interval. The coupling interval was defined differently depending on the initiation category. It was taken as the interval between the first beat of VT and the previous beat for sudden-onset initiation. In case of exatrasystolic initiation, it was defined as the interval between the first VPC and the previous beat.

### Clinical information

Clinical data for each patient including age, gender, underlying heart disease, left ventricular ejection fraction, and antiarrhythmic drug usage at the time of index arrhythmia was documented by review of clinical records.

### Statistical analysis

Continuous data are expressed as mean±SD. Means among different groups were compared by the analysis of variance. In case of nominal or ordinal data the groups were compared by using the chi-squire test. A P value < 0.05 was considered statistically significant. A possible relation between various clinical variables and the different modes of initiation were evaluated by logistic linear regression. Data analysis was performed by SPSS 13.0 (SPSS Inc., Chicago, IL, USA).

## Results

### Baseline characteristics

A total of 92 patients had ICD devices implanted between January 1999 and June 2001 at our institution. In this group of patients, 378 episodes of VT occurred among 65 patients. Of these, 182 episodes among 50 patients met study criteria; of 196 disqualified episodes, 90 had < 5 beats before the initiation of VT, 50 had no available EGM during VT for review, 19 had received no therapy for VT, 16 were polymorphic in nature, 10 episodes were pace-induced and 11 episodes were result of VF treatment by ICD. The mean age of patients in our study was 43±19 years with a minimum of 11 years and a maximum of 78 years. There were 40 men and 10 women in the group. The underlying heart disease was coronary artery disease (20 of 50 patients), arrhythmogenic right ventricular dysphasia (ARVD) (10 patients), idiopathic dilated cardiomyopathy (14 patients), heart tumor (2 patients), congenital heart disease (2 patients), and long QT syndrome (2 patients). The mean left ventricular ejection fraction for the group studied was 38±15%. All the patients were taking antiarrhythmic drug therapy at the time of index VT. Table 1 shows baseline characteristics of the study population.

### Arrhythmia characteristics

**Figure 1:**
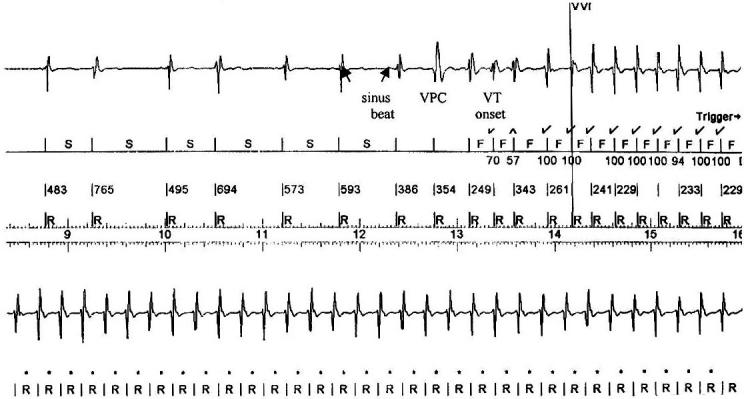
Initiation of VPC-induced sustained VT by a single VPC that is different in morphology from the VT

**Figure 2:**
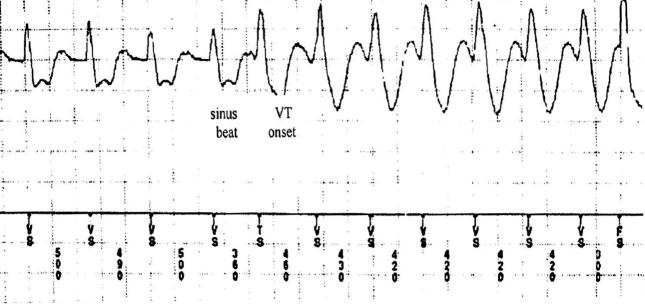
Sudden-onset monomorphic VT preceded by a single VPC that is similar in morphology to the VT

The most frequent initiation pattern was VPC-induced (Figure 1), observed in 106 episodes (58%), and followed by 76 episodes (42%) of VT with sudden onset initiation (Figure 2). Among the VPC-induced group, VPCs in 85 episodes (80%) were different in morphology from the subsequent VT. In only 21 of 106 episodes (20%), the premature depolarizations preceding the VT were of the same morphology. Table 2 lists the arrhythmia characteristics and clinical variable of the 2 main initiation categories.

Ten patients had only one episode of VT, whereas 40 patients had = 2 episodes. Among the 40 patients with multiple episodes, 35(88%) had at least 2 events with the same initiation sequence, while 14 (34%) had the same single pattern of initiation repeated during all subsequent episodes of VT.

The overall mean prematurity index was 0.71± 0.56 for all episodes. The sudden-onset group had a prematurity index of 0.71± 0.3, whereas the VPC-induced group had a prematurity index of 0.67±0.15. There was no statistically significant difference in prematurity index among the 2 initiation groups before and after controlling for underlying heart disease, antiarrhythmic drug usage, or degree of left ventricular dysfunction.

**Figure 3:**
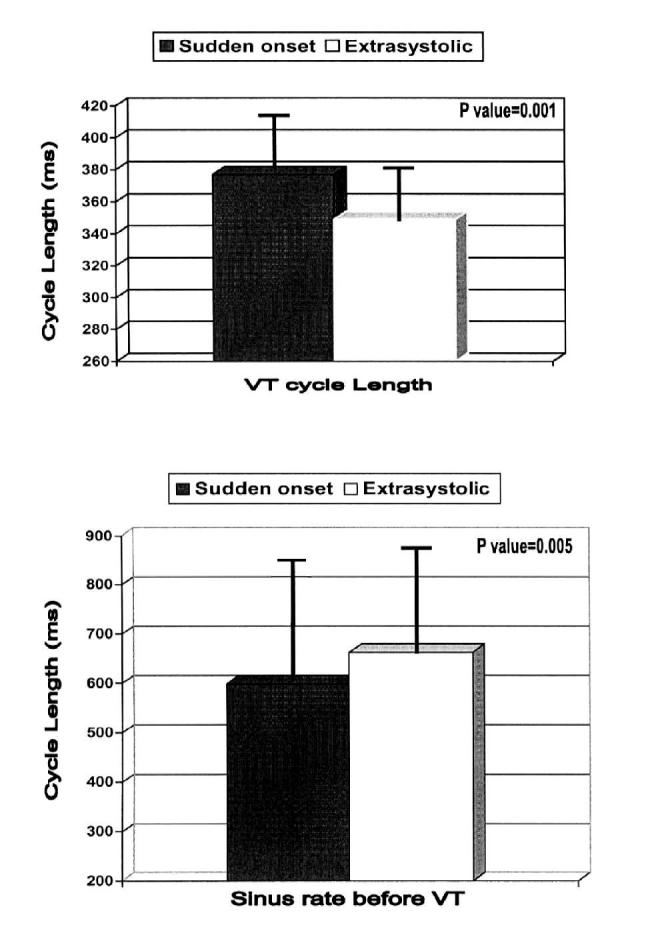
Relation of VT cycle length and preceding sinus rate to different VT initiation sequences. VT episodes following sudden-onset (black bars) initiation are slower than extrasystolic (open bars) onset (upper panel). Sinus rate is faster before sudden-onset than extrasystolic onset (lower panel)

The cycle length of sudden onset VT was slower than that of VT following VPC (Figure 3). The mean cycle length for VT with a sudden-onset initiation pattern was 377±30ms compared with 349±29 extrasystolic onset (P=0.001). The mean sinus rate was slower in the extrasystolic onset group (664 ±213ms) than the sudden-onset group (529±227ms) and the difference was statistically significant at P= 0.005.

### Clinical variables

Clinical parameters from each patient were analyzed for any possible association with modes of initiation (Table 2). There was no association of gender, underlying heart disease, age, ejection fraction and use of various types of antiarrhythmic drugs with an initiation sequence of monomorphic VT. The mean age in sudden onset group of patients was 37±18 years whereas in the extrasystolic initiation group it was 44±17 years and the difference was not statistically significant with P= 0.5. The mean ejection fraction was 37±16% in the sudden-onset VT and 36 ±13% in the extrasystolic onset group (P= 0.7)

### ICD therapy

The mean number of treatment applied by ICD to treat each episode of VT was 2.4±0.1 in the extrasystolic onset group and 2.3±0.2 in the sudden-onset group (P=0.5). The overall success rate was 93% for first therapy and 85% for second to sixth therapy. The success rate was calculated in each group of VT initiation separately and there was no association between different patterns of VT initiation and the success rate in a particular level of treatment (Table 2).

## Discussion

Our findings demonstrate that most VT episodes were initiated by VPCs. These were often different in morphology from the subsequent VT. The coupling interval was typically long. These findings are in agreement with the data of Marchlinski et al [16-18] Roelke et al [19], and Saeed et al [20] who studied mostly patients with coronary artery disease and previous myocardial infarction. They found a similar incidence and prematurity of VPCs before VT in their patient population. Mayerfeldt et al [21] also investigated the mode of the onset of VT in a group of patients with dilated cardiomyopathy and coronary artery disease. In their study single premature beats or couplets initiated three fourths of VT episodes.

A significant percentage of VT episodes started suddenly without a preceding VPC. Other investigators have classified these as VT with single VPC of similar morphology [19]. Like Saeed et al, we chose to assign these episodes to the VPC-induced (extrasystolic) category if VPC was loosely coupled or to the sudden-onset category if it was tightly coupled to the subsequent VT. This classification was adopted because the presence of tightly coupled and morphologically similar VPC was thought to represent a distinct electrophysiologic mechanism.

### Electrical and clinical differences between two VT initiation groups

Our study showed that the sudden-onset and extrasystolic initiation patterns tend to have different electrical characteristics. Sudden-onset VTs had longer cycle lengths and faster sinus rates before VT, where as VPC-initiated episodes had shorter cycle lengths and a slower sinus rate. These finding did not seem to be due to differences in the electrical (eg, coupling interval, prematurity index) or clinical (eg, heart disease, ejection fraction, or antiarrhythmic drug usage) variables among patients.

Other studies have shown that there is a high intraindividual reproducibility of VT with the same onset seen in up to 88% of patients with recurrent VT [20-22]. Our data are consistent with the observation of other investigators, showing 88% reproducibility of the initiation sequence among our patients. This highlights patient-specific factors, namely, the presence of fixed or functional anatomic pathways are important in the genesis of recurrent monomorphic VT.

Our study showed no correlation between VT initiation pattern and left ventricular systolic function. In contrast, other studies have revealed that sudden-onset VT is more common among patient with relatively preserved systolic function and VPC-initiated VT had poor left ventricular function (<35%) [20]. This difference may be related to the younger age of our patients and the inclusion of more cases of non-coronary heart disease in our study (with better left ventricular systolic function). Although we did not find any differences in initiation among the various disease categories, this could be related to the small sample size of the patients with coronary artery disease in our study. To the best of our knowledge, our study demonstrated for the first time that there is no association between pattern of VT initiation and the success rate of electrical therapy (antitachycardia pacing or direct-current cardioversion).

### Electrophysiologic implications

Although our study was an observational one and did not address the mechanism of VT initiation directly, several inferences can be made. In our study, VPCs that are often different in morphology and loosely coupled to subsequent tachycardia likely initiated VT via setting up reentry. VT may also start suddenly: (1) this may be due to a VPC arising from a site very close to the reentrant circuit or concealed decremental conduction of the sinus beat just before the VT setting up a reentry [3]; (2) sudden-onset initiation could also be due to a focus of increased automaticity or triggered activity. The first mechanism may be more important in patients with coronary artery disease, whose sinus beat may conduct slowly through diseased tissue to setup reentry, and the first beat exiting the circuit is actually the first beat of VT as evidenced by close coupling and similar morphology.

ICD tiered therapy was successful similarly in two VT-initiation groups. Thus, there is no need for different programming of antitachycardia pacing and direct-current cardioversion in two modes of VT-initiation. In addition, large data bases of these events may be helpful in answering clinical questions regarding disease or gender specificity of ventricular arrhythmias.

### Limitations

Visual inspection of the intracardiac electrogram is subjective and carries inherent limitations in interpreting morphologhy. Electrograms that appear similar may not originate from the same ventricular site. Also, all premature depolarizations were assumed to be ventricular in origin and the fact that some of them might have been supraventricular premature depolarization cannot be excluded.

## Conclusion

Dissimilarities between VT initiation patterns could not be explained by differences in electrical (coupling interval, and prematurity index) or clinical (heart disease, ejection fraction, and antiarrhythmic drug) variables among the patients. There is no association between pattern of VT initiation and the success rate of electrical therapy.

## Figures and Tables

**Table 1:**
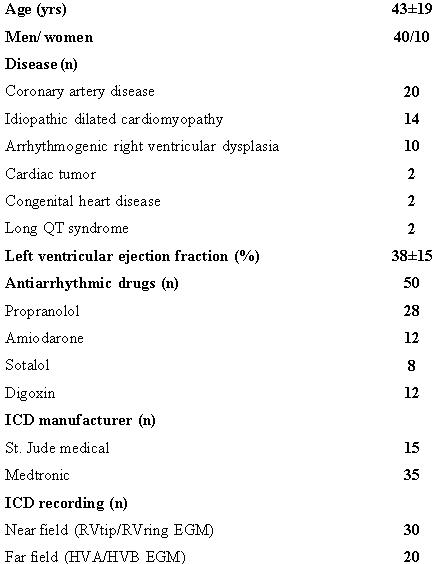
Baseline characteristics of study population (n=50)

**Table 2:**
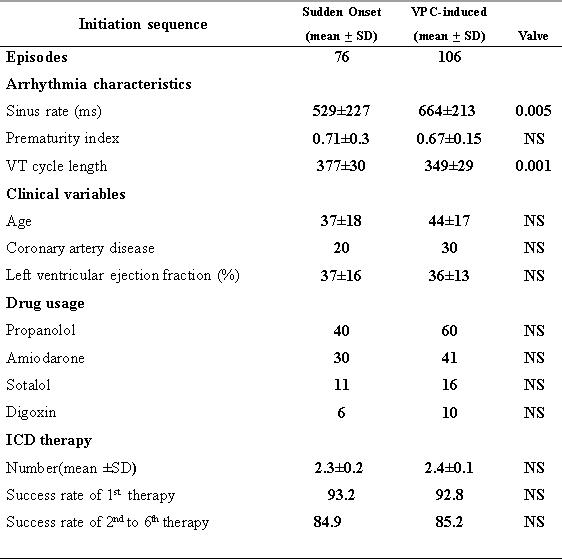
Arrhythmia characteristics and clinical variables of the two main VT initiation subgroups
